# Implementation of Cancer Plans in the United States: A Review

**DOI:** 10.3390/healthcare9030291

**Published:** 2021-03-07

**Authors:** Michael W. Bacchus, Bobbie McKee, Clement K. Gwede, Christopher R. Cogle

**Affiliations:** 1 Division of Hematology and Oncology, Department of Medicine, College of Medicine, University of Florida, Gainesville, FL 32610, USA; michaelbacchus@ufl.edu; 2 H. Lee Moffitt Cancer Center, Tampa, FL 33612, USA; Bobbie.Mckee@moffitt.org (B.M.); Clement.Gwede@moffitt.org (C.K.G.)

**Keywords:** health policy, cancer prevention, cancer control

## Abstract

State cancer plans facilitate prioritization and stakeholder engagement in preventing and controlling cancer. Implementation plans further help stakeholders prioritize efforts, reduce redundancy, and find opportunities for work synergies. A review of cancer plan implementations plans was performed in the development of an implementation plan for the Florida Cancer Plan. This review sought to identify, characterize, and summarize the use of implementation plans that support comprehensive cancer control activities. Although 100% of states and territories published a cancer plan and 78% of states provided funding for implementing their state cancer plans, only 32% published an implementation plan. Commonalities and unique features of state cancer plan implementations are presented and discussed. An example implementation plan is provided for states without a plan to model.

## 1. Introduction

A crucial component of comprehensive cancer control (CCC) is a plan developed collaboratively by multiple and diverse stakeholders with aspirational goals, evidence-based strategies, and measurable objectives [1,2,3]. Historically, each state prioritizes their goals, strategies, and objectives based on several factors including specific cancer burdens affecting their communities, stakeholder capacity, feasibility, and political will [4]. A typical cancer plan can contain twelve to sixty objectives, but usually encompasses the continuum of cancer from prevention to early detection to treatment to survivorship. As limited resources preclude working on all objectives at once, cancer consortiums must adopt some method of selecting objectives [5]. This selection process and work to synergize efforts makes up a state’s cancer control implementation plan. In some cases, an implementation plan document is crafted and used as an operational supplement to the cancer plan document.

The history of CCC plans and supplemental implementation plans within the United States represents a gradual but concerted effort to streamline cancer burden reduction into coordinated action. Early initiatives in CCC focused on the use of plans, in conjunction with registries, to target specialized foci, such as screening initiatives, incidence reduction, and mortality improvements [6,7,8]. At the federal level, the Center for Disease Control and Prevention (CDC) established the National Breast and Cervical Cancer Early Detection Program in 1991 in partnership with states’ departments of health to provide low-cost screening and diagnostic services to vulnerable populations [9]. The CDC furthered its cancer control programming the following year through the establishment of the National Program of Cancer Registries, which sought to characterize the current cancer burden at the federal level [9]. In 1998, the CDC launched the National Comprehensive Cancer Control Program (NCCCP) at a limited scale within select states [10]. Defined by a primary mission goal of promoting health equity through cancer control, the NCCCP grants resources and funding to relevant organizations to reduce cancer burden. Over the next two decades, the program successfully expanded to include all fifty states and various jurisdictions, each with well-defined and ambitious CCC plans [11]. The creation of dedicated implementation plans to supplement the overall CCC plan and increase the actualization of objectives, however, has been much more limited in scale.

Recently, the Florida Cancer Control and Research Advisory Council (formerly known as Cancer Control and Research Advisory Board or CCRAB) led the State of Florida in establishing its 2020–2025 Florida Cancer Plan [12]. The Council also established an Implementation Plan for the State of Florida’s Cancer Plan. In preparation for implementing the Florida Cancer Plan, the Council closely reviewed cancer plan implementation in other states to determine best practices. This review of published literature sought to identify, characterize, and summarize the use of implementation plans as a means of comprehensive cancer control by 50 States and 9 territories. Included in this analysis is a review of the common and unique elements of each implementation plan, and how such efforts synergize with existing cancer burden reduction initiatives. This review is a summary of the Council’s background research and is provided with the hopes of assisting other states, territories, and the public when designing and actuating their plans to ultimately reduce cancer burden.

## 2. Methods

**Figure 1:**
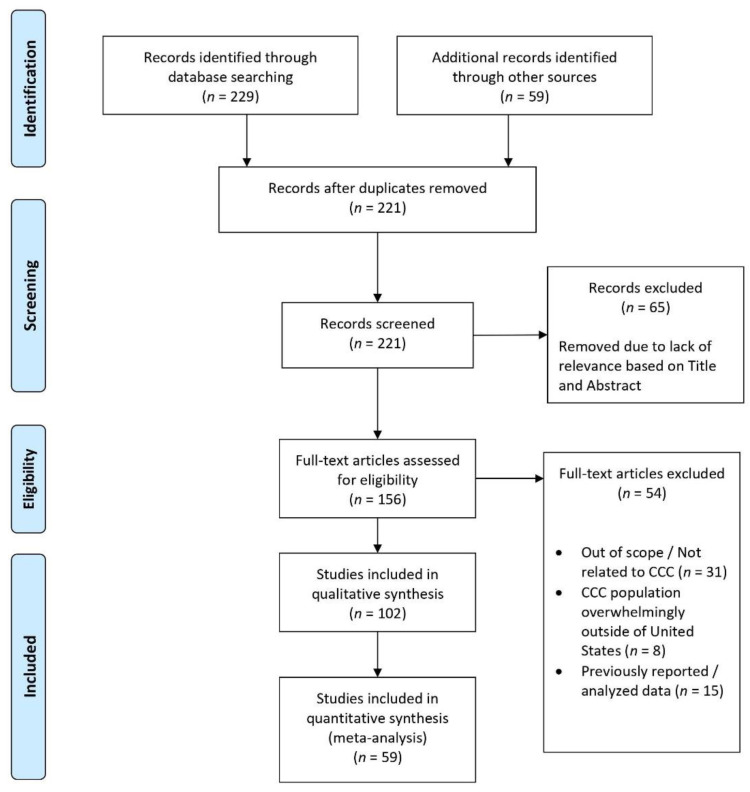
Flow diagram of search and study selection.

A review of published English articles was conducted using the guidelines established by the Preferred Reporting Items for Systematic Reviews and Meta-Analysis (PRISMA) [13]. A comprehensive literature search was conducted from June 2020–August 2020 through the PubMed-NCBI database using the following search terms / phrases: state cancer plan, cancer implementation plan, cancer control plan, and comprehensive cancer control. Additional studies were also collected using the references discussed within the collected articles. This study is exempt from IRB approval because the data used are derived from previously published research. Additionally, the official Department of Health websites for each state and jurisdiction were reviewed from June 2020–August 2020 to identify and review published NCCCP plans and implementation plans. Search and article criteria included specific discussions or reviews of cancer burden reduction from publications from 1990 and onwards. Figure 1 summarizes the search yield and study selection process using the standardized PRISMA flowchart. A total of 221 articles were screened and 156 were fully assessed, with 54 articles excluded from qualitative synthesis due to the content discussed being predominantly unrelated to CCC within the United States or repeated data. Fifty-nine (59) articles were used directly from published CCC plans for the quantitative analysis presented in Table 1 and Table 2. Appendix B includes the PRISMA checklist.

Study eligibility was determined a priori and included all publications that discussed comprehensive cancer control and/or implementation plans. Our analysis was guided by PICO (population, intervention, comparison, and outcome) principles of systematic reviews when applicable. The scope (population) of this review was defined as the states, territories, and jurisdictions associated with the United States. The intervention was the existence of published cancer control implementation plans or organizations. As a core component of this study was to compare the commonalities and differences of implementation plans (and identify regions that lacked such a plan), the comparison group was reviewing cancer control strategies between States that used a formalized implementation plan. A secondary function was comparing differing strategies among States with a cancer implementation plan versus States that do not have a plan. The outcome of this review is to provide a qualitative review of the distribution and defining features of cancer implementation plans that are produced by States and related jurisdictions.

Data extraction was performed independently by the first author (MWB) under the direct supervision of the senior author (CRC). Any disagreements were discussed and revised by the authors. Information was extracted on the characteristics of comprehensive cancer control, the use and design of cancer plans, funding to support implementation, and the use and design of implementation plans. All the data supporting the statements of this manuscript are presented and appropriately referenced throughout this document. We acknowledge the risk of selection and publication bias when screening and including articles, and the inherent difficulty in limiting such biases when the information used to form this study involves qualitative assessment to characterize plan elements.

Percentages were calculated from this data in the context of 59 states and territories. All descriptive statistics were analyzed using Microsoft Excel. Characteristics of comprehensive cancer control and plans were summarized using simple descriptive statistics which serve as the primary summary measurement.

## 3. Results

Every U.S. state (*N* = 50) and U.S. territory (*N* = 9) had an online publication of their cancer plan, with 46 of 59 (78%) publishing a current plan and 13 of 59 (22%) publishing an out-of-date plan (Table 1). The organization of NCCCP plan objectives varied from state to state and included specific categories such as screening and vaccination goals, as well as specific improvements in cancer survivorship quality of life [14,15,16].

Of the 59 states and territories, 19 (32%) had a distinct implementation plan document that specifically addressed cancer plan evidence-based implementation strategies. State departments of health participated in funding 46 of 59 (78%) implementation efforts.

The implementation plans took many forms. Nineteen states and territories published documents that presented implementation on a website. As an example, the State of Florida’s cancer plan implementation plan is provided in Appendix A. Nevada published “action plans” to correspond with task force objectives.

Nineteen states had websites that presented implementation strategies and served as a hub to link cancer stakeholders and initiatives, such as on Florida’s Department of Health website. Other states, such as Colorado and 14 others, directly solicited for implementation grant applications aligning with their cancer plans.

Collectively, these implementation plans demonstrate a wide variety of efforts by states and territories to actualize their state cancer plans.

## 4. Discussion

### 4.1. Common Elements among the Implementation Plans

Task forces, subcommittees, and workgroups for implementing state cancer plans were used in 34 states. These organizational structures functioned to organize and mobilize stakeholders. The Pennsylvania Cancer Coalition, for example, had workgroups focused on specific types of cancer such as lung and colorectal cancer [17]. Comparably, organizations such as Delaware’s Cancer Consortium had action teams dedicated to overarching topics such as risk reduction, prevention, and cancer registry [18]. Thirteen states (19%) used workgroups and coalitions based on regional model. In this strategy, states were divided into smaller regions based on city or population distribution to facilitate more compartmentalized approaches. Both topical task forces and regional coalitions provided targeted approaches to delegate responsibility within the broader cancer control plan and, when used in conjunction with an implementation plan, served as a vehicle for evidence-based actionable change. These findings are summarized in Table 2.

### 4.2. Unique Examples of Implementation

There were several instances for state cancer councils to use tailored strategies in their implementation plans. For example, New Mexico and Montana both had workgroups dedicated to cancer control in their Native American populations [19,20]. With the third and fifth largest proportion of Native American populations, respectively, the existence of dedicated workgroups facilitated initiatives that targeted healthcare disparities specific to Native Americans [21].

Focus on pediatric cancer was found in Idaho, which published a childhood cancer strategic plan alongside their comprehensive control plan during the 2006–2010 period [22]. Florida’s state cancer plan 2020–2025 also contained a pediatric cancer section and plans for implementation to include survivorship issues such as transition from adolescent care clinics to adult care clinics [6].

The Cancer Council of the Pacific Islands has sought to unify cancer control initiatives within the Pacific region through the creation a regional plan with the participating members of American Samoa, Commonwealth of the Northern Mariana Islands, Federated States of Micronesia, Guam, Republic of the Marshall Islands, and Republic of Palau. The result was a comprehensive plan in 2012 of constituent jurisdictions that sought to unify individual approaches under a broader framework [22]. Each of the individual jurisdictions also produced their own cancer control plans, and the existence of the additional regional comprehensive plan served to supplement individual efforts [23]. The Pacific Region plan also presented a tailored approach to cancer control that appreciates the nuances of the constituent demographics.

Geographically, states with implementation plans were distributed sporadically throughout the mainland United States except for the Southern United States, where only Florida and South Carolina had implementation plans. The reason for the notable absence of implementation plans is not clear and may involve other health priorities competing for health policy work.

### 4.3. Measuring Progress of Implementation

The core function of an implementation plan is to stimulate and maximize the evidence-based efforts made by stakeholders to complete the varied cancer control plan goals. To fully appreciate the effectiveness of these interactions and better guide resource allocation, states must also have systems to review cancer control progress. Most directly, the progress made in reducing cancer burden can be examined within each newly published cancer plan. Cancer plans set goals for over the course of a designated period (often five years) and are subsequently followed by a new plan with revised objectives. Thus, the production and release of regularly updated cancer plans inform individuals on the progress that has been made in the reduction in cancer burden, as well as discuss newly identified areas of focus. Further, cancer consortiums and affiliated organizations have participated in the distribution of burden reduction data online, as seen in New York and the Comprehensive Cancer Control Plan Dashboard website [24]. The dashboard actively tracks and displays forty pertinent measurements of cancer burden including prevention, detection, and survivorship, and provides comparisons between the most recent state data with previous baselines and their upcoming 2023 objectives. Through the aggregation of cancer control data presented in an accessible and regularly updated format, such dashboards provide a transparent review of the progress being made towards cancer control. The dashboards also inform policymakers on areas of deficiencies that require more attention to improve progress.

### 4.4. Synergies in Implementation

Collectively, these implementation plans enable resource allocation and accountability. The ability to measure the exact impact of an implementation plan and its synergy with the control plan remains difficult to quantify. States’ cancer registries function as an effective way to examine population-adjusted rates in specific cancer incidence, morality, and survival. Further, prior reports present the utility of general state cancer plans in actualizing burden reduction goals through increased communication and coordination among stakeholders [25]. However, attempts to attribute the degree of change in these statistics in states with an implementation plan compared to states that do not have an implementation plan are inherently confounded, as every area has varying demographics, culture, and evolving goals. Further, the absence of a published implementation plan did not imply that the resources for implementation were not readily accessible to stakeholders; these resources may have been distributed privately by the various cancer consortiums.

In the absence of an adequate control group to quantify the benefit of implementation plan, there may be qualitative benefits in documenting an implementation plan. One of the benefits of producing an implementation plan document is that it requires stakeholders to agree on elements for prioritizing collaborative work and then identifies those priorities for action. The Implementation Plan for the State of Florida is provided in Appendix A as an example of a model that could be adapted in states that do not presently have an implementation plan. Florida’s cancer plan implementation plan may also be used to update implementation plans in other states. Another benefit of producing a physical document for cancer plan implementation is that it encourages stakeholders to sign up for participation and enables a steering council to see areas of redundancy and complementary areas of synergy.

## 5. Conclusions

The evolution and expansion of the NCCCP was critical in establishing coordinated and standardized approaches to cancer reduction within the United States. This was accomplished through the organization of resources, stakeholders, and seed funding. The multifaceted nature of comprehensive cancer control at the state and local level is very challenging because of limited resources. This challenge can be greatly reduced through the production of implementation plans; however, such plans, in any form, are still absent in many US states and jurisdictions.

The implementation of a cancer plan through published documents, informational websites, funding opportunities, and task forces represents many of the fundamental approaches taken to reduce cancer burden. Equally important are methods to record and assess the extent of progress being made in cancer control, with updated cancer plans and online dashboards serving as transparent tools to present measured data.

The 19 implementation plans listed in Table 1 and their components described in Table 2 exemplify varied and innovative approaches to address specific cancer burden objectives and function as valuable reference for those interested in CCC. The linked websites in Table 1 also serve as a useful and powerful directory for individuals and organizations interested in exploring, establishing, and adopting their own CCC implementation plans. The collective identification of current cancer implementation plans and recognition of their utility in cancer control is critical for increased adoption of such plans, especially in states and regions that do not presently have a formalized implementation strategy. The increased production and adoption of implementation plans serve to further reduce cancer burden and supplement the existing efforts accomplished by cancer control plans. Implementation plans provide for the actionable evidence-based strategies to effectively execute Cancer Plan objectives.

This report also shows evidence for the significant and impactful work by the CDC in supporting US states and jurisdictions in planning their cancer prevention, early detection, and control. Capitalizing on this success, the CDC should receive more support for its work in assisting US states and jurisdictions to boost financial resources for implementing their cancer plans.

## Figures and Tables

**Table 1:** State Cancer Plans in the United States and Plans for Implementation, all access 31 August 2020.

State	Cancer Plan Website	**Implementation Plan**
**Alabama**	https://www.alabamapublichealth.gov/cancercontrol/ (accessed on 31 August 2020)	
**Alaska**	http://dhss.alaska.gov/dph/Chronic/Pages/Cancer/partnership/default.aspx (accessed on 31 August 2020)	
**Arizona**	https://www.azdhs.gov/prevention/tobacco-chronic-disease/cancer-prevention-control/az-cancer-coalition/index.php#az-cancer-control-prevent (accessed on 31 August 2020)	
**Arkansas**	https://arcancercoalition.org/about-us/ (accessed on 31 August 2020)	X
**California**	https://www.cdph.ca.gov/Programs/CCDPHP/DCDIC/CDSRB/Pages/California-Dialogue-on-Cancer-(CDOC).aspx (accessed on 31 August 2020)	
**Colorado**	https://www.coloradocancercoalition.org/colorado-cancer-fund/grant-application/ (accessed on 31 August 2020)	X
**Connecticut**	http://ctcancerpartnership.org/ (accessed on 31 August 2020)	
**Delaware**	https://www.healthydelaware.org/Consortium#:~:text=The%20Delaware%20Cancer%20Consortium%20was,potential%20methods%20for%20reducing%20both (accessed on 31 August 2020)	
**Florida**	http://www.ccrab.org/cancer-plan (accessed on 31 August 2020)	X
**Georgia**	https://dph.georgia.gov/cancer-prevention-and-control/comprehensive-cancer-control-program (accessed on 31 August 2020)	
**Hawaii**	https://health.hawaii.gov/cancer/home/coalition/ (accessed on 31 August 2020)	
**Idaho**	https://www.ccaidaho.org/about-ccai (accessed on 31 August 2020)	
**Illinois**	https://www.ipha.com/news/illinois-cancer-partnership#gsc.tab=0 (accessed on 31 August 2020)	
**Indiana**	https://www.in.gov/isdh/28395.html (accessed on 31 August 2020)	X
**Iowa**	https://canceriowa.org/grants/fy2021/ (accessed on 31 August 2020)	X
**Kansas**	http://cancerkansas.net/Workgroups (accessed on 31 August 2020)	
**Kentucky**	https://www.kycancerc.org/ (accessed on 31 August 2020)	
**Louisiana**	https://healthylouisiana.org/about (accessed on 31 August 2020)	
**Maine**	https://mainecancer.org/apply-for-a-grant (accessed on 31 August 2020)	
**Maryland**	https://phpa.health.maryland.gov/cancer/cancerplan/Pages/collaborative.aspx (accessed on 31 August 2020)	
**Massachusetts**	https://www.mass.gov/service-details/massachusetts-comprehensive-cancer-prevention-and-control-network-mccpcn-work (accessed on 31 August 2020)	
**Michigan**	https://www.michigancancer.org/CancerPlan/ComprehensiveCancerControlPlan-2016–2020.html (accessed on 31 August 2020)	
**Minnesota**		
**Mississippi**	https://www.umc.edu/cancerinstitute/Cancer-Research/Cancer-Registries/Mississippi%20Cancer%20Registry/Mississippi-Partnership-for-Comprehensive-Cancer-Control.html (accessed on 31 August 2020)	
**Missouri**	https://www.cancernmo.org/about-the-consortium (accessed on 31 August 2020)	X
**Montana**	https://dphhs.mt.gov/publichealth/cancer/cancercoalition (accessed on 31 August 2020)	X
**Nebraska**	https://www.necancer.org/implementation (accessed on 31 August 2020)	
**Nevada**	https://nevadacancercoalition.org/get-involved/task-force (accessed on 31 August 2020)	X
**New Hampshire**	https://www.nhcancerplan.org/index.php/workgroups/93-task-forces/221-goals-objectives-strategies (accessed on 31 August 2020)	
**New Jersey**	https://www.nj.gov/health/ces/public/surveillance-unit/ (accessed on 31 August 2020)	X
**New Mexico**	http://www.nmcancercouncil.org/ (accessed on 31 August 2020)	
**New York**	https://www.health.ny.gov/diseases/cancer/consortium/index.htm (accessed on 31 August 2020)	
**North Carolina**	https://publichealth.nc.gov/chronicdiseaseandinjury/cancerpreventionandcontrol/index.htm (accessed on 31 August 2020)	
**North Dakota**	http://www.ndhealth.gov/compcancer/cancer-programs-and-projects/nd-comprehensive-cancer-control-sub-contract-request-for-proposals/ (accessed on 31 August 2020)	X
**Ohio**	https://www.ohiocancerpartners.org/ (accessed on 31 August 2020)	
**Oklahoma**	https://www.ok.gov/health/Disease,_Prevention,_Preparedness/Chronic_Disease_Service/Cancer_Prevention_Programs_/Comprehensive_Cancer_Control_Program/Oklahoma_Comprehensive_Cancer_Network_(OCCN)/index.html (accessed on 31 August 2020)	
**Oregon**	https://www.oregon.gov/oha/PH/DiseasesConditions/ChronicDisease/Documents/hpcdp-strategic-plan.pdf (accessed on 31 August 2020)	
**Pennsylvania**	http://www.pacancercoalition.org/workgroups (accessed on 31 August 2020)	X
**Rhode Island**	https://www.prcri.org/our-partners (accessed on 31 August 2020)	
**South Carolina**	https://www.sccancer.org/the-alliance/ (accessed on 31 August 2020)	X
**South Dakota**	https://www.cancersd.com/resources/grant-opportunities/ (accessed on 31 August 2020)	X
**Tennessee**	https://www.tn.gov/health/health-program-areas/fhw/tccc/coalition-leadership.html (accessed on 31 August 2020)	
**Texas**	https://www.cprit.state.tx.us/about-us (accessed on 31 August 2020)	X
**Utah**	http://www.ucan.cc/members/implementation/ (accessed on 31 August 2020)	X
**Vermont**	http://vtaac.org/our-partnerships/ (accessed on 31 August 2020)	
**Virginia**	https://cancercoalitionofvirginia.org/pages/about-CACV.php (accessed on 31 August 2020)	
**Washington**	https://www.doh.wa.gov/YouandYourFamily/IllnessandDisease/Cancer/ComprehensiveCancerControl#:~:text=The%20Washington%20State%20Comprehensive%20Cancer,broad%20spectrum%20of%20cancer%20issues (accessed on 31 August 2020)	X
**West Virginia**	https://moh.wv.gov/awards-mini-grants/Pages/default.aspx (accessed on 31 August 2020)	X
**Wisconsin**	https://wicancer.org/programs/action-areas/ (accessed on 31 August 2020)	X
**Wyoming**	https://health.wyo.gov/publichealth/prevention/cancer/wyoming-cancer-coalition/ (accessed on 31 August 2020)	
**Washington, D.C.**	https://www.dccanceranswers.org/about/mission/ (accessed on 31 August 2020)	X
**Pacific Region**	http://www.pacificcancer.org/index.html (accessed on 31 August 2020)	
**America Samoa**	http://www.pacificcancer.org/pacific-partners/american-samoa.html (accessed on 31 August 2020)	
**Commonwealth of Northern Mariana Islands**	http://www.pacificcancer.org/pacific-partners/northern-mariana-islands.html (accessed on 31 August 2020)	
**Federated States of Micronesia**	http://www.pacificcancer.org/pacific-partners/federated-states-of-micronesia.html (accessed on 31 August 2020)	
**Guam**	http://www.pacificcancer.org/pacific-partners/guam.html (accessed on 31 August 2020)	
**Republic of the Marshall Islands**	http://www.pacificcancer.org/pacific-partners/marshall-islands.html (accessed on 31 August 2020)	X
**Republic of Palau**	http://www.pacificcancer.org/pacific-partners/palau.html (accessed on 31 August 2020)	
**Puerto Rico**	https://www.iccp-portal.org/puerto-rico-model-collaborative-and-parcipative-approach (accessed on 31 August 2020)	

**Table 2:** Analysis of State Cancer Plans in the United States.

State or Territory	Is the Cancer Plan Current to 2020/2021?	Engagement: Existence of Task Forces and/or Workgroups	Existence of Regional Coalitions	Grant Applications on Website
Alabama	X	X		
Alaska	X	X		
Arizona				
Arkansas	X			X
California				
Colorado	X	X		X
Connecticut		X		
Delaware	X	X		
Florida	X		X	
Georgia			X	
Hawaii	X	X	X	
Idaho	X	X		
Illinois	X			
Indiana	X	X	X	X
Iowa	X			X
Kansas	X	X	X	
Kentucky	X		X	
Louisiana	X	X	X	X
Maine	X	X		X
Maryland	X			
Massachusetts	X	X		
Michigan	X	X		
Minnesota	X	X	X	
Mississippi	X		X	
Missouri	X			
Montana	X	X		X
Nebraska	X	X		
Nevada	X	X		
New Hampshire	X	X		
New Jersey			X	
New Mexico		X		
New York	X	X		
North Carolina	X	X		
North Dakota	X	X		X
Ohio	X	X		
Oklahoma	X			
Oregon				
Pennsylvania	X	X		X
Rhode Island		X		
South Carolina	X	X		X
South Dakota	X	X		X
Tennessee	X	X		
Texas		X		X
Utah	X			X
Vermont	X	X		
Virginia	X		X	
Washington	X	X	X	
West Virginia	X	X	X	X
Wisconsin	X	X		X
Wyoming	X	X		
Pacific Region		X	X	
America Samoa				
Commonwealth of Northern Mariana Islands				
Federated States of Micronesia	X			
Guam	X	X		
Republic of the Marshall Islands	X			
Republic of Palau	X			
Puerto Rico		X		
Washington DC	X			

## Data Availability

Not applicable.

## Appendix A. Florida Cancer Plan 2020–2025 Implementation Plan

**Purpose**

The Florida Cancer Plan is a guide for assuring a data- and stakeholder-driven strategy to reduce Florida’s cancer burden. The key to this plan lies in its successful implementation. CCRAB believes that effective implementation of the Florida Cancer Plan must ensure that efforts are:

- Coordinated and collaborative
- Non-duplicative
- Leveraging the strengths of individuals and organizations involved in cancer control efforts
- Addressing gaps in efforts
- Measurable and progress is tracked
- Utilizing existing, in-kind, and new resources

Because of limited resources, not all objectives in the Florida Cancer Plan can be worked on immediately or simultaneously. The Florida Cancer Plan’s priority objectives will be selected by CCRAB in close collaboration with the Regional Cancer Collaboratives and other state cancer control stakeholders. Priorities will be determined using criteria that consider need and impact, feasibility, likelihood for success, and interest in working on the issue. CCRAB, the Regional Cancer Collaboratives, and other state cancer control stakeholders will select evidence-based strategies in this plan that correspond to the priority objectives and cooperatively develop action plans for each of the strategies. *Please note:* It is recommended that CCRAB and the Collaboratives jointly select priority objectives. CCRAB members will develop action plans for statewide implementation and Collaboratives will develop action plans for regional implementation. It may be that some Collaboratives will want to select additional objectives from the plan to work on in their region, based on regional priorities and needs; however, there will be greater synergy and potential outcomes if CCRAB and the Collaboratives are all working on the same priority objectives.

**Selecting Priorities from the Cancer Plan for Implementation**

Every 1–2 years CCRAB will engage the Regional Collaboratives and other key stakeholders in a process to identify priority objectives from the Florida Cancer Plan for implementation and to select strategies associated with those priorities to work on, using a written action plan to guide efforts. Choosing priorities at the objective level will enable CCRAB and stakeholders to focus on measurable outcomes to achieve together.

Guidance/Criteria for Selecting Objectives

The following criteria will be used to help guide selection of priority objectives:

- **Importance:**
  ⚬ Is it important that Floridians achieve this objective over the next 1–2 years?
  ⚬ Is the objective a sentinel or bellwether for change?
- **Effectiveness:**
  ⚬ Is this objective the most useful effort we can make to achieve the goal?
  ⚬ Will achieving this objective lead to a meaningful impact on Florida’s cancer burden?
- **Measurable:**
  ⚬ Are reliable data available now or could data be developed to measure outcomes?
- **Equitable:**
  ⚬ If the objective is met, to what degree would all Floridians benefit?
- **Synergistic:**
  ⚬ Are stakeholders willing to work on this objective?

Guidance/Criteria for Selecting Strategies

The following criteria will be used to select priority strategies from the Florida Cancer Plan in association with the priority objectives:

- **Evidence-Based:**
  ⚬ Is the strategy based on research or proven best practices, thus increasing the likelihood that the strategy will be successful? (Note: The strategies in the Plan should be evidence-based, as this criterion was used for inclusion; however, it is good to re-examine the strategy to ensure it is the best approach, given available evidence.)
- **Feasibility:**
  ⚬ Is it feasible to execute the strategy over the next 1–2 years, considering the costs associated, resources required, cultural appropriateness, political will, likelihood of stakeholders working cooperatively, etc.?
- **Synergistic:**
  ⚬ Is this strategy one we need to accomplish together, rather than one stakeholder bearing sole responsibility?
  ⚬ Are stakeholders willing to work on this strategy?

Once the strategies are selected, CCRAB and the Collaboratives will develop written action plans for each priority objective and share them with the key stakeholders. The action plan will serve as a guide for all stakeholders working collaboratively on a priority objective. Action plans should include (see Action Plan Template below):

- Priority objective and measures (from the Florida Cancer Plan)
- Priority strategies chosen to achieve the objective (from the Florida Cancer Plan)
- Tasks or activities to achieve the strategies with relevant timeframes for completion and who is responsible
- Short- and/or intermediate-term outcomes with measures to gauge progress on achieving strategies (if needed)
- Resources and information needed to achieve strategies (funding, in-kind)
- Stakeholders to engage (who are the key stakeholders to engage and how will we do that, etc.)
- Communication processes (who do we need to communicate with about the strategy and when, etc.)
- Progress notes section

Resources for Plan Implementation

CCRAB does not have funding to implement the Florida Cancer Plan and it is not a financial fiduciary for the Plan, i.e., CRAB does not receive funds from stakeholders for implementation. However, CCRAB members and other stakeholders do have opportunities to commit and/or leverage resources (funding and in-kind resources) to implement the Plan, such as their own organizational resources. Identifying and securing the resources needed to implement the priority objectives and strategies is a critical task for CCRAB, the Regional Cancer Collaboratives and other stakeholders to take in order to successfully implement priorities.

**Stakeholder Engagement in Plan Implementation**

CCRAB

CCRAB is made up of 15 members representing 15 cancer stakeholder organizations. CCRAB serves as the steering body for implementing the Florida Cancer Plan. At each of CCRAB’s biennial meetings, it will review the state’s progress toward plan objectives by reviewing available data relative to each objective’s baseline and target. During CCRAB biennial meetings special attention will be given to progress being made on the current priority objectives and the action plans developed for each strategy chosen under that objective. There may be a need to convene additional meetings of CCRAB members and other stakeholders to discuss collaborative efforts and communicate about progress and opportunities related to the priority objectives and strategies.

Florida Department of Health and Regional Cancer Collaboratives

As a CCRAB member, the Florida Department of Health (DOH) is instrumental in collecting data for measuring progress towards several of the Plan objectives. The DOH will also implement many of the strategies in the Plan to achieve the stated objectives. The Florida Department of Health uses CDC support to coordinate the activities of six Regional Cancer Collaboratives. The Collaboratives are essential in implementing many of the Plan’s strategies.

During the six regional Town Hall meetings conducted to provide input on the Florida Cancer Plan 2020-2025, Regional Cancer Collaborative members indicated they were willing to work with CCRAB on Plan implementation and expressed a desire to increase 2-way communication between CCRAB and the Collaboratives. Engagement of the Collaboratives is a critical part of the Goal 1 of the cancer plan: *To maximize cancer control resources by increasing collaboration among Florida cancer control stakeholders.*

Suggested strategies to engage the Regional Cancer Collaboratives from the Florida Cancer Plan, with additional actions are:

- Increase the number and diversity of Floridians engaged in the Regional Cancer Collaborative activities.
  ⚬ Encourage Floridians interested in joining the fight against cancer to contact their local Regional Cancer Collaborative.
  ⚬ Highlight the work of the Collaboratives on the CCRAB website and in progress reports and other communications.
- Encourage Regional Cancer Collaborative members and stakeholders to use the Florida Cancer Plan for planning, funding, and advocacy.
  ⚬ Meet with the leaders of the six Regional Cancer Collaboratives on an annual basis to get their input on priority objectives, share progress on priorities, identify gaps/opportunities to work together on, and to strategize about coordinated efforts.
  ⚬ Disseminate priority objective information to Collaboratives so they can work to align their regional efforts with statewide efforts.
  ⚬ Communicate overall plan successes, progress, and areas of continued need among Collaboratives periodically (e.g., quarterly) throughout the year.
  ⚬ Create a CCRAB speakers list of members willing to attend regional Collaborative meetings to share CCRAB information/updates and hear about Collaborative efforts.
- Coordinate with Regional Cancer Collaboratives to use consistent and accurate cancer control messages.
  ⚬ Work with the FL DOH and Collaboratives to identify cancer control messages that are/will be associated with priority objectives identified from the plan. For each priority objective, identify possible public and provider education and advocacy messages that can be shared with the Collaboratives.
  ⚬ Provide written materials/messages to Collaboratives that they may disseminate and adapt for use in their own regions.
- Work with the Regional Cancer Collaboratives to identify two areas for focused collaborative efforts over the 5-year plan period
  ⚬ Conduct an initial meeting with the FL DOH and leadership of the six Collaboratives to identify potential areas of collaborative focus for the 5-year plan period (**Note**: Feedback gathered during the six regional Town Hall meetings in 2019 indicated that the following may be areas of interest: transportation, lung cancer screening, and HPV vaccination).
  ⚬ Use input on areas of interest from Collaboratives to help guide selection of priority objectives from the cancer plan for the first 1–2 years of implementation.
  ⚬ During subsequent annual meetings with Collaborative leadership, set aside time to review progress and adjust strategies for the focused collaborative efforts.
  ⚬ Communicate with Collaboratives on a regular (e.g., quarterly) basis to coordinate campaign efforts, via email and/or conference call.

Additional Stakeholders

Florida has a rich environment of cancer control stakeholders within Florida’s many communities, clinics, hospitals, and boardrooms. Success of achieving the goals and objectives of this plan depend greatly on cancer control stakeholders across the state working together to coordinate and collaborate on cancer control efforts.

Additional statewide and regional partner engagement will be needed to support priority objective implementation efforts. For each priority objective CCRAB should identify potential stakeholders (beyond CCRAB members and Regional Cancer Collaboratives) to engage in the statewide effort. As action plans are developed, stakeholders should be identified, with a strategy for engaging them: e.g., who is best to contact the stakeholder, who do we know within the stakeholder organization, what is our “ask” and what is our follow-up plan.

CCRAB should also identify processes to respond to inquiries from stakeholders interested in partnering with CCRAB on Plan implementation efforts. For example, the CCRAB Executive Director and Chair could receive and then triage inquiries from interested stakeholder organizations and ensure follow-up occurs.

CCRAB may consider convening an annual summit of Florida cancer control stakeholders to foster communication and collaborative efforts to achieve cancer plan objectives. This could occur in conjunction with an already established state conference or meeting, where CCRAB can present Plan progress. Or CCRAB may co-convene an already established state conference, where CCRAB member organizations and other cancer control stakeholders may be in attendance.

**Tracking Progress with Plan Implementation**

Achieving progress requires measurement. CCRAB will measure progress toward the priority objectives by regularly examining cancer-relevant data. This examination process will consist of the following:

Track Progress in Achieving Priority Objectives and Associated Strategies

- Each priority objective selected from the Florida Cancer Plan has measures associated with it. CCRAB should collect and report on those measures to gauge progress. Additional short- or intermediate-term outcomes may need to be identified to gauge progress for each priority objective.
- Tracking and sharing progress on the statewide collaboration among CCRAB, Regional Cancer Collaboratives, and other state cancer control stakeholders is also important. For each Action Plan, metrics for collaboration should be included to convey what can be achieved by working together. For example, under an objective to increase the proportion of limited stage (Stage 1 and 2) lung cancer and decrease late-stage (Stage 3 and 4) lung cancer in Florida, strategies may include increasing awareness, access, and usage of lung cancer screening in target populations. Metrics for collaboration may include tracking the amount of time between action steps, diversity of resources used, and/or comparing the individual stakeholder’s projected value in the effort (number of lung cancer patients diagnosed with limited stage cancer versus late-stage cancer) versus the collaboration’s realized value of the effort demonstrating synergy.

Tracking Overall Plan Progress

- Reporting on all Florida Cancer Plan goals and associated objectives/measures should be included in the state statute-mandated annual report from CCRAB to the Florida Governor and Florida Legislature (s. 1004.435, section 4(p)), with special focus on Plan priority goals and objectives.

Communicating Progress

- A Florida Cancer Plan 2020-2025 dashboard may be an effective way to communicate progress. A dashboard that includes all plan objectives/measures with a special focus on the priority objectives chosen from the Florida Cancer Plan may be the most effective way to communicate progress, to call attention to successes, and indicate if more collaborative action is needed to achieve intended outcomes).
- CCRAB’s annual reports could include easy-to-understand graphical depictions of overall progress on priority objectives, including what CCRAB, the FL DOH, the Regional Cancer Collaboratives and other key stakeholders are doing/were able to achieve together.
- Annual progress summaries (1–2 pagers with easy to understand graphics) on each priority objective would be good resources to share with Regional Cancer Collaboratives and other stakeholders, to convey successes and outline additional resources/collaborative efforts needed to achieve objectives.

**Implementation Plan Timeline and Next Steps**

By the April 2020 CCRAB meeting:

- Finalize this implementation plan with input from the FL DOH and Regional Collaborative staff and leadership and CCRAB members

At the April 2020 CCRAB meeting:

- Select priority objectives from the plan using the criteria outlined above (if possible, Regional Collaborative staff and leaders should attend this meeting, to provide input on selecting strategies)
- Identify a process and timeframe for CCRAB to select strategies within the priority objective and complete an action plan
- Discuss next steps for Collaboratives, including development of action plans for their region
- Identify key stakeholder communication strategies and timeframes, e.g., how and how often to communicate with Regional Collaboratives on plan implementation

After the April 2020 CCRAB meeting:

- Finalize and disseminate CCRAB priority objective action plans to CCRAB members and other stakeholders, as appropriate
- Regional Collaboratives will hold meetings to select strategies for the agreed upon priority objectives, that leverage existing strengths, programs, efforts within their region, and identify regional stakeholders to recruit to help with implementation
- Communicate with the FL DOH, Regional Collaboratives and other key stakeholders on a regular basis about Florida Cancer Plan implementation progress, needs and next steps (e.g., monthly calls with Collaborative staff and leadership, periodic priority objective updates to all CCRAB members and other stakeholders)
- Establish a timeframe for systematically reviewing progress on the priority objectives (every 1-2 years) and selecting to continue and/or identify new 1-2 year priorities from the Plan

**Florida Cancer Plan: Action Plan Template**

**Priority objective and measures (from the Florida Cancer Plan)**								
**Priority Strategies Chosen to Achieve the Objective (from the Cancer Plan)**	**Tasks to Achieve the Strategy**	**Timeframe for Completion**	**Person, Organization Responsible**	**Short- and Intermediate-Term Outcomes, Measures**	**Resources, Information Needed**	**Stakeholders to Engage**	**Communication Processes**	**Progress Notes**
Strategy 1	Task 1 Task 2 Task 3							
Strategy 2	Task 1 Task 2 Task 3							

## Appendix B. PRISMA Checklist

**Section/Topic**	**#**	**Checklist Item**	**Reported on Page #**
**TITLE**			
Title	1	Identify the report as a systematic review, meta-analysis, or both.	1
**ABSTRACT**			
Structured summary	2	Provide a structured summary including, as applicable: background; objectives; data sources; study eligibility criteria, participants, and interventions; study appraisal and synthesis methods; results; limitations; conclusions and implications of key findings; systematic review registration number.	1
**INTRODUCTION**			
Rationale	3	Describe the rationale for the review in the context of what is already known.	1–2
Objectives	4	Provide an explicit statement of questions being addressed with reference to participants, interventions, comparisons, outcomes, and study design (PICOS).	1–2
**METHODS**			
Protocol and registration	5	Indicate if a review protocol exists, if and where it can be accessed (e.g., Web address), and, if available, provide registration information including registration number.	2–3
Eligibility criteria	6	Specify study characteristics (e.g., PICOS, length of follow-up) and report characteristics (e.g., years considered, language, publication status) used as criteria for eligibility, giving rationale.	2–3
Information sources	7	Describe all information sources (e.g., databases with dates of coverage, contact with study authors to identify additional studies) in the search and date last searched.	2–3
Search	8	Present full electronic search strategy for at least one database, including any limits used, such that it could be repeated.	2–3,
Study selection	9	State the process for selecting studies (i.e., screening, eligibility, included in systematic review, and, if applicable, included in the meta-analysis).	2–3
Data collection process	10	Describe method of data extraction from reports (e.g., piloted forms, independently, in duplicate) and any processes for obtaining and confirming data from investigators.	2–3
Data items	11	List and define all variables for which data were sought (e.g., PICOS, funding sources) and any assumptions and simplifications made.	2–3
Risk of bias in individual studies	12	Describe methods used for assessing risk of bias of individual studies (including specification of whether this was done at the study or outcome level), and how this information is to be used in any data synthesis.	2–3
Summary measures	13	State the principal summary measures (e.g., risk ratio, difference in means).	2–3
Synthesis of results	14	Describe the methods of handling data and combining results of studies, if done, including measures of consistency (e.g., I^2^) for each meta-analysis.	2–3
Risk of bias across studies	15	Specify any assessment of risk of bias that may affect the cumulative evidence (e.g., publication bias, selective reporting within studies).	2–3
Additional analyses	16	Describe methods of additional analyses (e.g., sensitivity or subgroup analyses, meta-regression), if done, indicating which were pre-specified.	2–3
**RESULTS**			
Study selection	17	Give numbers of studies screened, assessed for eligibility, and included in the review, with reasons for exclusions at each stage, ideally with a flow diagram.	3–5
Study characteristics	18	For each study, present characteristics for which data were extracted (e.g., study size, PICOS, follow-up period) and provide the citations.	3–5
Risk of bias within studies	19	Present data on risk of bias of each study and, if available, any outcome level assessment (see item 12).	3–5
Results of individual studies	20	For all outcomes considered (benefits or harms), present, for each study: (a) simple summary data for each intervention group (b) effect estimates and confidence intervals, ideally with a forest plot.	3–5
Synthesis of results	21	Present results of each meta-analysis done, including confidence intervals and measures of consistency.	3–5
Risk of bias across studies	22	Present results of any assessment of risk of bias across studies (see Item 15).	3–5
Additional analysis	23	Give results of additional analyses, if done (e.g., sensitivity or subgroup analyses, meta-regression [see Item 16]).	N/A
**DISCUSSION**			
Summary of evidence	24	Summarize the main findings including the strength of evidence for each main outcome; consider their relevance to key groups (e.g., healthcare providers, users, and policy makers).	5–9
Limitations	25	Discuss limitations at study and outcome level (e.g., risk of bias), and at review-level (e.g., incomplete retrieval of identified research, reporting bias).	5–9
Conclusions	26	Provide a general interpretation of the results in the context of other evidence, and implications for future research.	9–10
**FUNDING**			
Funding	27	Describe sources of funding for the systematic review and other support (e.g., supply of data); role of funders for the systematic review.	10
